# Digital epistemological beliefs as predictors of academics’ digital technology literacy

**DOI:** 10.3389/fpsyg.2026.1848263

**Published:** 2026-06-11

**Authors:** Elvan Gökçen, Eyüp İzci

**Affiliations:** 1 Department of Child Development, Social Sciences Vocational School, Harran University, Şanlıurfa, Türkiye; 2 Department of Curriculum and Instruction, Faculty of Education, Inonu University, Malatya, Türkiye

**Keywords:** academics, digital epistemological belief, digital information, digital technology literacy, higher education

## Abstract

**Introduction:**

The aim of this study was to examine the relationship between academics’ digital epistemological beliefs and their digital technology literacy and to determine the extent to which digital epistemological beliefs predict digital technology literacy.

**Methods:**

The study was conducted with 673 academics employed at public universities in Türkiye. Data were collected using the Digital Epistemological Belief Scale, the Digital Technology Literacy Scale, and a personal information form developed by the researcher. The data were analyzed using descriptive statistics, Pearson correlation analysis, simple linear regression, independent samples t-test, and one-way analysis of variance (ANOVA). Prior to the analyses, normality assumptions were tested through arithmetic mean, median, kurtosis, and skewness values.

**Results:**

The findings indicated that academics’ perceptions of digital epistemological beliefs and digital technology literacy were at a moderate level. No significant differences were found in digital epistemological beliefs, digital technology literacy, or their sub-dimensions according to gender. However, significant differences were identified based on academic title, professional seniority, and academic field. Furthermore, a strong, positive, and statistically significant relationship was found between digital epistemological beliefs and digital technology literacy. The results also demonstrated that digital epistemological beliefs significantly predicted digital technology literacy.

**Discussion:**

The findings suggest that stronger digital epistemological beliefs are associated with higher levels of digital technology literacy among academics. The study contributes to the literature by providing a comprehensive examination of the relationship between these two constructs through the use of original, valid, and reliable measurement instruments.

## Introduction

Digital transformation, driven by advancements in the internet and technology, has profoundly altered societal structures and daily life (Gencer and Aktan, 2021), creating a pressing need for individuals to adapt to the technological age (Metin, 2011). Digitalization has had particularly significant impacts in the field of education, transforming the speed and methods of accessing information and promoting the widespread use of technological tools in teaching and learning processes (Başaran et al., 2020). Today, the use of technological tools such as computers, projectors, and the internet has increasingly replaced traditional classroom tools, a trend that has accelerated with the proliferation of interactive whiteboards (Polat and Özcan, 2014).

The widespread integration of computers into education aims to equip young individuals with computer literacy and prepare them for professional life (Karhan, 2007). Access to and use of digital knowledge are closely linked to the concept of epistemology, as it raises fundamental questions about how knowledge is obtained and the nature of knowledge itself (Deryakulu, 2004). Epistemological beliefs shape individuals’ perspectives on knowledge and the learning process (Hofer, 2001), serving as a cognitive filter through which learning is interpreted and internalized (Demir, 2009).

While traditional epistemology views knowledge as certain and unchanging—acquired through observation and experience (Çamur, 2016) contemporary epistemological perspectives argue that knowledge is dynamic and constructed (Schommer, 1990). Developments in the information age have compelled educators to enhance their epistemological awareness, which in turn influences their perspectives on the use of technology in instructional processes (Akpınar and Akyıldız, 2013). Accordingly, educational curricula are increasingly incorporating content aimed at developing technology literacy (Huyugüzel-Çavaş, 2009).

Technology literacy has become a top priority in the educational systems of many countries today (Huyugüzel-Çavaş, 2009). New teaching methods and techniques are being designed to enhance students’ technological competencies. In this context, educators are responsible for helping students acquire skills in accessing, evaluating, and applying knowledge to solve problems (Karhan, 2007). As the integration of technology in education continues to grow, educators’ epistemological beliefs and conceptions of knowledge play a critical role in shaping their attitudes toward technology use (Akpınar and Akyıldız, 2013).

Technology literacy is recognized as an essential skill for individuals to participate effectively in the digital world. Educators are expected to guide students in developing a critical perspective toward technology, enabling them to make informed, knowledge-based decisions (Huyugüzel-Çavaş, 2009). Advanced epistemological beliefs contribute to individuals’ ability to consciously access and utilize technological knowledge (Schommer, 1990).

In conclusion, digital transformation and epistemological beliefs are key to developing technology literacy. Beliefs about knowledge and learning influence how individuals adapt to technology and shape educational change. Therefore, educators must consciously manage this process to equip students with essential digital skills (Karhan, 2007). Enhancing epistemological awareness is essential for increasing technology literacy and for using technology effectively in education.

Following the identification of the first COVID-19 case in Turkey on March 11, 2020, formal education was suspended, and a transition to remote education began on March 23, 2020 (Budak and Korkmaz, 2020). Following the COVID-19 pandemic, the accelerated integration of digital technologies into educational processes has further increased the importance of technology-enhanced learning practices and online educational platforms. In particular, the widespread adoption of digital learning environments in higher education institutions has led researchers to investigate the effects of these technologies on the learning processes, academic achievement, and classroom engagement of students with diverse abilities and needs (Achtypi et al., 2026; Dobson Waters and Torgerson, 2021; Grover and Wright, 2023; McNicholl et al., 2019; Sandu and Taylor, 2025; Sumner et al., 2021). During this period, the rapid expansion of remote education highlighted the importance of digital tools and applications. Considering the likelihood of similar extraordinary circumstances in the future, it is anticipated that education may continue through distance learning models (Keskin and Özer, 2020). In this context, the hybrid learning model—which combines the advantages of both face-to-face and distance education—has emerged as a promising approach (Liu et al., 2020). Gnaur et al. (2020) examined the effectiveness of online learning during the pandemic through student feedback and emphasized the value of blended learning environments. In Turkey, the Ministry of National Education has also been planning for the implementation of hybrid education models. Within this evolving context, digital technology literacy has become a critical element of the education system.

The shift to distance learning has emphasized the need for strong digital technology literacy among educators and other stakeholders (Doğan and Yılmaz, 2013; Saracaloğlu et al., 2013). The COVID-19 pandemic underscored the growing integration of education and technology (Telli and Altun, 2020). As knowledge becomes increasingly digital, educators must develop digital pedagogical skills. Digital epistemological beliefs—recognized as part of 21st-century competencies—are now seen as essential for academics’ technological proficiency and pedagogical effectiveness.

This study offers an original contribution to the literature by examining the relationship between digital epistemological beliefs and digital technology literacy within the context of higher education through a sample of faculty members. While previous studies have generally addressed digital competence, technology integration, or digital literacy as separate constructs, the present study approaches the role of digital epistemological beliefs in shaping digital technology literacy from a more holistic perspective. Furthermore, the measurement instruments developed by the researcher constitute an additional contribution of the study, as they not only provide relational findings but also introduce valid and reliable new measurement tools to the literature. In addition, the large sample size and the focus on faculty members’ positions within digital transformation processes provide a contemporary and distinctive perspective on ongoing discussions regarding digitalization, technology integration, and digital competence in higher education.

Literature shows that the link between digital epistemological beliefs and digital technology literacy remains underexplored. Studies in this area can shed light on the readiness of Turkish higher education for the information age and support academics in curriculum and instructional design. In this regard, the main research question of the study was defined as:

“What is the nature of the relationship between academics’ digital epistemological beliefs and their digital technology literacy?”

To address this problem, answers were sought to the following sub-questions:

1. What is the perceived level of academics’ digital epistemological beliefs?
2. What is the perceived level of academics’ digital technology literacy?
3. Do academics’ digital epistemological beliefs significantly differ based on gender, academic title, years of experience, and field of study?
4. Do academics’ digital technology literacy levels significantly differ based on gender, academic title, years of experience, and field of study?
5. Is there a relationship between academics’ digital epistemological beliefs and their digital technology literacy?
6. Do academics’ digital epistemological beliefs significantly predict their digital technology literacy?

## Methods

This study was designed using quantitative research methods and employed both a correlational research model and a causal-comparative (ex post facto) design. The correlational model aims to determine the relationships between independent and dependent variables without any external intervention (Johnson and Christensen, 2019; Sönmez and Gülderen Alacapınar, 2013). This model evaluates how variables change together and the nature of this change under natural conditions (Karasar, 2011).

The causal-comparative model, on the other hand, investigates cause-and-effect relationships by examining the current situation without manipulating any variables (Sönmez and Gülderen Alacapınar, 2013; Sözbilir, 2014). In this model, the relationships between one or more categorical independent variables (e.g., gender, academic title, years of experience, field of study) and one or more quantitative dependent variables are analyzed (Johnson and Christensen, 2019). The correlational design was employed to examine the relationships between digital epistemological beliefs and digital technology literacy, whereas the causal-comparative design was used to identify differences according to demographic variables.

### Population and sample

In this study, participants were selected on a voluntary basis, and the sample was determined using the simple random sampling method. This approach, in which each unit has an equal probability of being selected, is referred to as simple random sampling (Büyüköztürk et al., 2009). Accordingly, the sample of the study consisted of 673 academics working at public universities in Turkey. An examination of the demographic characteristics of the participants reveals that 47.40% of the academics were female and 52.60% were male. Regarding academic titles, 23.48% held the title of Professor, 22.59% were Associate Professors, 28.53% were Assistant Professors, and 25.41% were Lecturers. In terms of professional experience, 12.04% of the academics had 0–5 years, 38.93% had 6–10 years, 25.56% had 11–15 years, 11.74% had 16–25 years, and another 11.74% had more than 26 years of experience. When categorized by academic field, 25.41% were employed in health sciences, 26.45% in social sciences, 25.71% in basic and engineering sciences, and 22.44% in sports and fine arts.

### Data collection instruments

The data collection process in the study covered the development and administration stages of the Digital Epistemological Belief Scale and the Digital Technology Literacy Scale. During the scale development process, the relevant theoretical framework and literature were first reviewed, and item pools were subsequently generated in line with expert opinions. Initially, a 51-item draft form was prepared for the Digital Epistemological Belief Scale, while a 67-item draft form was prepared for the Digital Technology Literacy Scale. The draft scales were submitted to field experts for content and face validity, and after the necessary revisions were made, they were reviewed in terms of language and clarity and made ready for pilot implementation.

Following ethics committee approval, the scale forms were distributed via Google Forms to faculty members working at state universities in Turkey through e-mail and online communication channels. Participation in the data collection process was voluntary; participants were informed about the purpose of the study and assured that the data obtained would be used solely for scientific purposes. Within the scope of the scale development study, data were collected from a total of 769 faculty members; data obtained from 324 participants were used for Exploratory Factor Analysis, while data from 445 participants were used for Confirmatory Factor Analysis.

Exploratory Factor Analysis (EFA) and Confirmatory Factor Analysis (CFA) were conducted on separate sample groups to examine the construct validity of the scales (*n* = 324 for EFA; *n* = 445 for CFA). The findings indicated satisfactory construct validity, as evidenced by high KMO values (0.941 and 0.982), significant Bartlett’s Test of Sphericity results (*p* < 0.001), strong factor loadings, and explained variance rates of 77.176 and 74.827%, respectively. Furthermore, the CFA results demonstrated excellent model fit indices for both scales (e.g., RMSEA = 0.013/0.018, CFI = 0.99, GFI = 0.95, SRMR = 0.024/0.022). In addition, high Cronbach’s alpha coefficients, together with Composite Reliability (CR) and Average Variance Extracted (AVE) values exceeding the recommended thresholds, supported the reliability and convergent validity of the scales (Gökçen, 2024).

The first section of the data collection form included a personal information form designed to determine participants’ gender, academic title, seniority, and field of specialization. The second section included the items of the Digital Epistemological Belief Scale and the Digital Technology Literacy Scale.

#### Digital Epistemological Belief Scale

The Digital Epistemological Belief Scale is in a five-point Likert format, scored between 1 and 5. The scale consists of a total of 24 items. It has 5 sub-dimensions (Accuracy of Digital Epistemology, Function of Digital Epistemology, Source of Digital Epistemology, Nature of Digital Epistemology, and Scope of Digital Epistemology). The sub-dimension Accuracy of Digital Epistemology includes 10 items, Function of Digital Epistemology includes 4 items, Source of Digital Epistemology includes 5 items, Nature of Digital Epistemology includes 3 items, and Scope of Digital Epistemology includes 2 items. To examine the overall reliability of the scale, Cronbach’s alpha reliability analysis was conducted, and the result was 0.948. The Cronbach’s alpha values for the sub-dimensions were: Accuracy of Digital Epistemology 0.954; Function of Digital Epistemology 0.930; Source of Digital Epistemology 0.881; Nature of Digital Epistemology 0.913; and Scope of Digital Epistemology 0.892 (Gökçen, 2024).

#### Digital Technology Literacy Scale

The Digital Technology Literacy Scale is in a five-point Likert format, scored between 1 and 5. The scale consists of a total of 25 items. It has 2 sub-dimensions (Necessity of Digital Technology and Self-Efficacy). The sub-dimension Necessity of Digital Technology includes 5 items, and the sub-dimension Self-Efficacy includes 20 items. To examine the overall reliability of the scale, Cronbach’s alpha reliability analysis was conducted, and the result was 0.976. The Cronbach’s alpha values for the sub-dimensions were: Necessity of Digital Technology 0.928 and Self-Efficacy 0.982 (Gökçen, 2024).

### Data analysis

Initially, normality assumptions were tested for the collected data. In this context, arithmetic mean, median, kurtosis, and skewness coefficients were examined. As a result of the analyses, it was determined that the data were normally distributed. Following the confirmation of normality assumptions, parametric tests were used to address the sub-problems of the research. Accordingly, in addition to descriptive statistics, Pearson correlation, simple linear regression, independent samples t-test, and one-way analysis of variance (ANOVA) were applied.

To address the sixth sub-problem of the study, “Do academics’ digital epistemological beliefs predict their digital technology literacy?,” a regression analysis was conducted. Prior to the regression analyses, the assumptions of normality, autocorrelation, and multicollinearity were examined. The normality of the data was evaluated based on mean–median proximity as well as skewness and kurtosis values, and all values were found to be within the acceptable range of ±2 (George and Mallery, 2010). In addition, Durbin–Watson values between 1 and 3 indicated the absence of autocorrelation, while VIF values below 10 demonstrated that there was no multicollinearity problem (Field, 2005). Accordingly, it was concluded that the assumptions required for the regression analyses were satisfied.

The regression equation regarding the sixth sub-problem of the study, which examines whether academics’ perceptions of digital epistemological beliefs predict their perceptions of digital technology literacy, is given below:

Digital Technology Literacy = 0.761 + 0.715 × Digital Epistemological Belief.

The regression equation regarding the sixth sub-problem of the study, which examines whether the sub-dimensions of digital epistemological beliefs predict academics’ perceptions of digital technology literacy, is presented below:

Digital Technology Literacy = 0.710 + 0.173 × Source + 0.149 × Nature + 0.275 × Accuracy + 0.167 × Function.

In the Likert-type scales used in the study, an interval of 0.8 points was utilized to interpret the participants’ response levels. These intervals correspond to the following levels:

“Very Low” (1.00–1.80), “Low” (1.81–2.60), “Moderate” (2.61–3.40), “High” (3.41–4.20), and “Very High” (4.21–5.00).

## Findings and interpretation

### Findings and interpretation regarding academics’ perceived levels of digital epistemological beliefs

In this section of the study, the findings related to the sub-problem, “What is the perceived level of academics’ digital epistemological beliefs?,” which was formulated in line with the purpose of the research, are presented in Table 1.

**Table 1:** Descriptive results regarding academics’ perceived levels of digital epistemological beliefs.

Variables	$\bar{X}$	*S*
Digital epistemological belief	3.11	0.90
Source of digital epistemology	3.22	1.08
Nature of digital epistemology	3.10	1.29
Scope of digital epistemology	3.00	1.33
Accuracy of digital epistemology	3.08	1.18
Function of digital epistemology	3.09	1.21

According to Table 1, academics’ perceived levels of digital epistemological beliefs were found to be moderate. When the sub-dimensions were examined, it was also observed that the perceived levels of the source, nature, scope, accuracy, and function of digital epistemology were all at a moderate level.

In a study conducted by Settaşı (2022), it was found that teachers working in schools affiliated with the Ministry of National Education generally had moderate epistemological beliefs. Similarly, studies conducted by Akyıldız (2014), Kurt (2009), and Karhan (2007) also reported comparable findings regarding both the sub-dimensions and overall results. However, a study by Şahin (2021) revealed that 478 teachers exhibited more developed epistemological beliefs.

Although epistemological beliefs are known to be resistant to change, global events have shown that such beliefs can be reshaped. The COVID-19 pandemic has underscored the necessity for adaptation in teaching and learning processes due to challenges encountered in the implementation of remote and hybrid education models. In this context, a study by Özeren (2020) concluded that high school teachers acknowledged the nature of knowledge but were unable to fully reflect this understanding in their instructional practices. The study emphasized the need to address epistemological deficiencies.

According to the findings, academics’ digital epistemological beliefs were at a moderate level. This may stem from factors such as educational background, experience, teaching methods, student profiles, and institutional support. Especially, limited technology use and inadequate institutional resources appear to hinder belief development.

### Findings and interpretation regarding academics’ perceived levels of digital technology literacy

In this section of the study, findings related to the second sub-problem, “What is the perceived level of academics’ digital technology literacy?,” which was formulated in accordance with the purpose of the research, are presented in Table 2.

**Table 2:** Descriptive results regarding academics’ perceived levels of digital technology literacy.

Variables	$\bar{X}$	*S*
Digital technology literacy	2.98	1.05
Necessity of digital technology	3.06	1.15
Self-efficacy	2.96	1.12

According to Table 2, the digital technology literacy levels of academics were found to be moderate. When the sub-dimensions were examined, the levels of necessity of digital technology and self-efficacy were also observed to be at a moderate level. In a study conducted by Minea-Pic (2020), it was emphasized that digital technology has transformed teacher learning in OECD countries and has become an essential element for teachers’ professional development. The OECD (2021) report stated that the COVID-19 pandemic made digital technologies vital for education, as the closure of schools shifted learning to online platforms, revealing a pressing need to enhance teachers’ digital literacy. In this process, the use of technology should be considered not merely as a compulsory solution, but as an integrated and sustainable component of educational processes (Mercader and Castro, 2026; Riva et al., 2021; Scully et al., 2021). Similarly, Yılmaz and Çakır (2022) found that preservice teachers had moderate levels of digital literacy. Usta and Korkmaz (2010) indicated that preservice teachers’ skills in using digital technologies are a critical factor affecting the quality of the education system. Furthermore, Çetin et al. (2012) noted that teachers’ positive attitudes toward digital technologies play a significant role in encouraging students to use these technologies effectively.

According to the findings of the present study, academics’ perceptions of digital technology literacy were determined to be at a moderate level. This result suggests that their skills may be influenced by their educational and professional experiences, technology use habits, and instructional approaches. Factors such as limited institutional support and resources and student characteristics may also contribute to the maintenance of a moderate level. Therefore, organizing digital literacy training programs for academics by universities may help enhance their perception levels.

### Findings and interpretation regarding academics’ perceptions of digital epistemological beliefs by gender, academic title, seniority, and field of study

In order to examine the third sub-problem of the study—whether academics’ perceptions of digital epistemological beliefs significantly differ according to the variables of gender, academic title, seniority, and field of study—independent samples t-test was used for comparisons involving two groups, and one-way ANOVA was used for comparisons involving more than two groups.

When Table 3 is examined, it is observed that the differences in academics’ digital epistemological beliefs and their sub-dimensions according to gender are not statistically significant (*p* > 0.05). Based on the research findings, gender does not appear to have a significant effect on digital epistemological beliefs and their sub-dimensions. Similarly, previous studies by Doğan (2019), Şahin (2021), Şaşmaz (2019), Olgun (2018), Kaya and Ekiçi (2017), Şenler and İrven (2016), Kazu and Erten (2015), Sadıç and Çam (2015), Adak and Bakır (2017), Sapancı (2012), Karabulut and Ulucan (2012), İzgar and Dilmaç (2008), and Deryakulu and Bıkmaz (2003) also found no significant differences in epistemological beliefs based on gender. This outcome is consistent with earlier studies that suggest both men and women possess similar epistemological beliefs.

**Table 3:** Independent samples *t*-test results regarding academics’ perceptions of digital epistemological beliefs by gender.

Scale and sub-dimensions	Gender	$\bar{X}$	*s*	*t*	*p*
Digital epistemological beliefs	Female	3.05	0.93	1.495	0.135
	Male	3.15	0.86		
Source of digital epistemology	Female	3.14	1.09	1.737	0.083
	Male	3.29	1.07		
Nature of digital epistemology	Female	3.06	1.30	0.775	0.439
	Male	3.14	1.28		
Scope of digital epistemology	Female	2.91	1.33	1.694	0.091
	Male	3.09	1.33		
Accuracy of digital epistemology	Female	3.03	1.20	0.946	0.344
	Male	3.12	1.16		
Function of digital epistemology	Female	3.04	1.25	0.903	0.367
	Male	3.13	1.17		

However, in some studies, the gender variable was found to yield significant differences. For instance, Eroğlu and Güven (2006), Kızıklı (2016), and Meral and Çolak (2009) identified significant gender-based differences in epistemological beliefs. Murat and Erten (2018) reported a positive effect in favor of male teachers, while Kurt (2009) found that female students had more advanced beliefs about the accuracy of knowledge. Ordu (2019) revealed that female teachers had more developed epistemological beliefs compared to their male counterparts. Mahasneh (2018), in a study investigating the relationship between university students’ concepts of teaching and learning and their epistemological beliefs, noted that female students had more opportunities to explore, discuss, and express themselves during the learning process. Similarly, Aslan (2017) found a significant gender difference in favor of females. Based on the results of such studies, it can be concluded that gender does not consistently lead to significant differences in epistemological beliefs.

When examining Table 4, it was determined that the differences in instructors’ digital epistemological beliefs and their sub-dimensions were statistically significant based on their academic titles (*p* < 0.01). *Post hoc* Scheffé test results revealed that the perceptions of instructors regarding “Digital Epistemological Beliefs,” “Source of Digital Epistemology,” “Nature of Digital Epistemology,” “Scope of Digital Epistemology,” “Accuracy of Digital Epistemology,” and “Function of Digital Epistemology” were higher among Professors and Associate Professors.

**Table 4:** ANOVA test results for academics’ digital epistemological belief perception levels according to academic title variable.

Scale and sub-dimensions	Title	$\bar{X}$	*s*	*F*	*p*
Digital epistemological belief	Professor	3.43	0.81	29.406	0.001*
	Assoc. Prof.	3.44	0.72		
	Asst. Prof.	2.82	0.90		
	Lecturer	2.83	0.92		
Source of digital epistemology	Professor	3.54	0.92	22.738	0.001*
	Assoc. Prof.	3.61	0.87		
	Asst. Prof.	2.92	1.13		
	Lecturer	2.92	1.13		
Nature of digital epistemology	Professor	3.42	1.12	10.753	0.001*
	Assoc. Prof.	3.38	1.19		
	Asst. Prof.	2.82	1.32		
	Lecturer	2.87	1.37		
Scope of digital epistemology	Professor	3.25	1.30	11.304	0.001*
	Assoc. Prof.	3.38	1.23		
	Asst. Prof.	2.77	1.30		
	Lecturer	2.70	1.36		
Accuracy of digital epistemology	Professor	3.44	1.13	18.462	0.001*
	Assoc. Prof.	3.41	1.07		
	Asst. Prof.	2.74	1.16		
	Lecturer	2.82	1.17		
Function of digital epistemology	Professor	3.39	1.24	12.341	0.001*
	Assoc. Prof.	3.37	1.12		
	Asst. Prof.	2.91	1.18		
	Lecturer	2.75	1.16		

*p < 0.01.

Şahin (2021), in a study conducted with teachers, found that academic title did not lead to a significant difference in epistemological beliefs. Similarly, Yılmaz (2014) reported that teachers and administrators had generally similar epistemological beliefs, although teachers scored higher in the sub-dimension of “belief in the role of effort in learning.” Arslan (2017) also concluded that academic title had no significant impact on epistemological belief levels and that all faculty members exhibited well-developed epistemological beliefs.

However, based on the findings of this study, it was observed that faculty members with the titles of Professor and Associate Professor demonstrated higher levels of digital epistemological beliefs and their sub-dimensions (source, nature, scope, accuracy, and function of digital epistemology). It can be argued that as academic title, seniority, and experience increase, individuals gain access to a wider range of information sources and develop a broader perspective on the nature and scope of knowledge. Moreover, as academic careers progress, the responsibility and desire to access validated and accurate information also increase, which may explain the significant difference in digital epistemological beliefs in relation to academic titles.

When Table 5 is examined, it is observed that there are statistically significant differences in faculty members’ digital epistemological beliefs and their subdimensions according to their professional seniority (*p* < 0.01). The Scheffe *post hoc* test conducted to determine the source of the differences revealed that faculty members with 16–25 years and 26 years or more of professional experience have higher levels of perception in terms of “Digital Epistemological Beliefs” and its subdimensions: “Source of Digital Epistemology,” “Nature of Digital Epistemology,” “Scope of Digital Epistemology,” “Accuracy of Digital Epistemology,” and “Function of Digital Epistemology.”

**Table 5:** ANOVA test results regarding faculty members’ digital epistemological belief perception levels by professional seniority variable.

Scale and sub-dimensions	Professional seniority	$\bar{X}$	*s*	*F*	*p*
Digital epistemological belief	0–5 years	2.79	0.92	8.763	0.001*
	6–10 years	3.06	0.90		
	11–15 years	3.03	0.89		
	16–25 years	3.44	0.77		
	26 years and above	3.43	0.86		
Source of digital epistemology	0–5 years	2.92	1.10	5.688	0.001*
	6–10 years	3.14	1.10		
	11–15 years	3.18	1.11		
	16–25 years	3.58	0.82		
	26 years and above	3.51	1.02		
Nature of digital epistemology	0–5 years	2.81	1.41	3.954	0.004*
	6–10 years	3.09	1.30		
	11–15 years	2.97	1.31		
	16–25 years	3.45	1.12		
	26 years and above	3.39	1.13		
Scope of digital epistemology	0–5 years	2.61	1.40	3.998	0.003*
	6–10 years	3.01	1.30		
	11–15 years	2.96	1.34		
	16–25 years	3.44	1.28		
	26 years and above	3.06	1.31		
Accuracy of digital epistemology	0–5 years	2.78	1.19	5.773	0.001*
	6–10 years	3.02	1.18		
	11–15 years	2.97	1.16		
	16–25 years	3.47	1.10		
	26 years and above	3.41	1.16		
Function of digital epistemology	0–5 years	2.71	1.13	5.588	0.001*
	6–10 years	3.04	1.16		
	11–15 years	3.06	1.23		
	16–25 years	3.21	1.23		
	26 years and above	3.57	1.24		

*p < 0.01.

In studies conducted by Şahin (2021), Özeren (2020), Kahraman (2020), Öner (2019), Usta (2019), and Murat (2018), professional seniority was found to cause significant differences. Karhan (2007) indicated that teachers with 1–5 and 6–10 years of seniority had higher epistemological beliefs than those with 26–30 years. However, Gül Biçer and Özgenel (2020) found no significant difference in school administrators’ epistemological beliefs based on professional seniority. Similarly, Kaya and Ekici (2017) and Güngör (2016) reported no significant differences in teachers’ epistemological beliefs based on seniority. Ordu (2019), on the other hand, found that teachers with lower seniority had more developed beliefs.

The findings of this study indicate that professional seniority creates a significant difference in digital epistemological beliefs and their subdimensions. Faculty members with 16–25 years and over 26 years of professional experience were found to have higher levels of perception in terms of digital epistemological beliefs and their subdimensions. Several factors may explain why faculty members with greater seniority possess stronger digital epistemological beliefs. Experience and accumulated knowledge—acquired through years of teaching and learning—enhance these beliefs. Moreover, the need to engage in continuous learning and integrate new information into instructional practices also contributes to the development of such beliefs. Therefore, it can be expected that faculty members with higher professional seniority have more advanced digital epistemological beliefs.

When Table 6 is examined, it is observed that the differences in faculty members’ digital epistemological beliefs and their sub-dimensions according to their academic fields are statistically significant (*p* < 0.01). According to the results of the *post hoc* Scheffé test conducted to determine the source of the differences, it was found that faculty members working in the fields of basic and engineering sciences have higher levels of perception regarding “Digital Epistemological Beliefs,” as well as the sub-dimensions of “Source of Digital Epistemology,” “Nature of Digital Epistemology,” “Scope of Digital Epistemology,” “Accuracy of Digital Epistemology,” and “Function of Digital Epistemology.”

**Table 6:** ANOVA test results of faculty members’ digital epistemological belief perception levels according to the field of study variable.

Scale and sub-dimensions	Field of study	$\bar{X}$	*s*	*F*	*p*
Digital epistemological belief	Health sciences	2.99	0.87	33.517	0.001*
	Social sciences	2.94	0.87		
	Basic and engineering sciences	3.65	0.71		
	Sports and fine arts	2.81	0.91		
Source of digital epistemology	Health sciences	3.11	1.09	27.745	0.001*
	Social sciences	3.09	1.09		
	Basic and engineering sciences	3.8	0.78		
	Sports and fine arts	2.82	1.1		
Nature of digital epistemology	Health sciences	2.92	1.3	16.188	0.001*
	Social sciences	3.02	1.25		
	Basic and engineering sciences	3.65	1.1		
	Sports and fine arts	2.77	1.33		
Scope of digital epistemology	Health sciences	2.86	1.35	16.547	0.001*
	Social sciences	2.81	1.32		
	Basic and engineering sciences	3.59	1.22		
	Sports and fine arts	2.72	1.26		
Accuracy of digital epistemology	Health sciences	2.98	1.19	18.653	0.001*
	Social sciences	2.84	1.13		
	Basic and engineering sciences	3.62	1.06		
	Sports and fine arts	2.83	1.17		
Function of digital epistemology	Health sciences	2.96	1.19	11.779	0.001*
	Social sciences	2.99	1.2		
	Basic and engineering sciences	3.53	1.05		
	Sports and fine arts	2.83	1.27		

*p < 0.01.

Schommer-Aikins (2004) compared university students’ epistemological beliefs with Biglan’s classification of academic disciplines and found similar correlations among epistemological beliefs regarding mathematics, social sciences, and business. With increased experience, some field-specific findings have also been observed. Doğan (2019) examined the relationship between science teachers’ epistemological beliefs and their awareness of STEM (Science, Technology, Engineering, and Mathematics), finding that science education graduates had more advanced epistemological beliefs than graduates of physics and chemistry education.

However, other studies by Kaya (2018), Akyıldız and Akpunar (2012), Kösemen (2012), Bacanlı-Kurt (2010), and Karhan (2007) did not find significant differences in teachers’ epistemological beliefs based on academic field. Özeren (2020) found that teachers’ epistemological beliefs showed significant differences in the sub-dimension of “Knowledge Generation Process” based on their academic discipline.

This study reveals that academic discipline significantly shapes digital epistemological beliefs. Faculty members in basic and engineering sciences scored higher across all sub-dimensions—such as source, nature, scope, accuracy, and function—likely due to their close engagement with digital tools, frequent technology use, and ongoing involvement with innovation. Additionally, the digital orientation of students in these fields may encourage faculty to develop stronger digital epistemological beliefs.

### Findings and discussion regarding faculty members’ perceptions of digital technology literacy in relation to gender, academic title, seniority, and academic discipline

In examining the fourth sub-problem of the study—whether faculty members’ perceptions of digital technology literacy significantly differ based on gender, academic title, years of professional experience (seniority), and academic discipline—an independent samples t-test was used for comparisons between two groups, and a one-way ANOVA was employed for comparisons among more than two groups.

As seen in Table 7, no statistically significant difference was found in faculty members’ digital technology literacy levels and its sub-dimensions based on gender (*p* > 0.05). In a study conducted by Yazıcıoğlu et al. (2020), it was also found that preservice teachers’ digital literacy levels did not differ significantly according to gender. Similarly, research by Ağırtaş and Çavuş (2022) found no significant gender-based differences in the level of digitalization among university faculty members. Other studies by Akkoyunlu and Soylu (2010), Baran et al. (2023), and Koç and Albayrak (2020) also observed no significant differences between faculty members’ gender and their digitalization status or technological proficiency. However, studies by Yılmaz and Çakır (2022) and Kıyıcı (2008) found that males had higher levels of digital literacy than females. Similarly, Arredondo-Trapero et al. (2021) also confirmed the influence of gender on the tendency to use digital technologies; however, their findings indicated that male participants were more likely to engage with technology-related issues.

**Table 7:** *T*-test results for faculty members’ digital technology literacy perception levels according to gender variable.

Scale and sub-dimensions	Gender	$\bar{X}$	*s*	*t*	*p*
Digital technology literacy	Female	2.96	1.07	−0.44	0.66
	Male	3.00	1.02		
Necessity of digital technology	Female	3.06	1.17	−0.027	0.978
	Male	3.06	1.13		
Self-efficacy	Female	2.94	1.15	−0.505	0.613
	Male	2.98	1.10		

The findings show that gender does not significantly affect perceptions of digital literacy and its sub-dimensions. This may be due to equal access to technology training, resources, and professional development opportunities across genders, as well as institutional and cultural efforts promoting gender balance.

When Table 8 is examined, it is seen that the differences in faculty members’ digital technology literacy levels and its sub-dimensions according to academic title variable are statistically significant (*p* < 0.01). The results of the Scheffe *post hoc* test, conducted to determine the source of these differences, show that faculty members’ perceptions of “Digital Technology Literacy,” “Necessity of Digital Technology,” and “Self-Efficacy” are higher among professors and associate professors.

**Table 8:** ANOVA test results of faculty members’ perceptions of digital technology literacy by academic title variable.

Scale and sub-dimensions	Title	$\bar{X}$	*s*	*F*	*p*
Digital technology literacy	Professor	3.15	0.99	8.01	0.001*
	Assoc. Prof.	3.23	0.98		
	Asst. Prof.	2.84	1.05		
	Lecturer	2.77	1.09		
Necessity of digital technology	Professor	3.21	1.11	11.285	0.001*
	Assoc. Prof.	3.43	1.03		
	Asst. Prof.	2.91	1.16		
	Lecturer	2.78	1.17		
Self-efficacy	Professor	3.14	1.08	6.02	0.001*
	Assoc. Prof.	3.18	1.06		
	Asst. Prof.	2.82	1.13		
	Lecturer	2.77	1.16		

*p < 0.01.

In a study by Ağırtaş and Çavuş (2022), it was found that the level of digitalization among faculty members did not significantly differ according to gender, but it did differ according to age. Faculty members aged between 31 and 40 demonstrated higher levels of technology use in social life compared to those aged 40 and above. These findings are supported by other studies, including those by Akkoyunlu (2002), Aksoy et al. (2021), Arslan (2019), Ataman (2016), Carrington and Robinson (2009), Kalemkuş (2016), Karakuş (2020), Marsh et al. (2017), and Mertoğlu (2020). However, Baran et al. (2023)reported that there was no significant difference between academics’ academic titles and their scores on the self-assessment scale of technological competence.

The differences observed in this study regarding faculty members’ digital technology literacy levels by academic title—particularly in favor of professors and associate professors—can be attributed to several factors. These faculty members are often more engaged in research and publication activities, which require intensive use of digital technologies. Furthermore, their increased involvement in leadership and administrative roles necessitates investment in and effective use of digital technologies. Greater participation in continuous professional development and educational opportunities may also contribute to the enhancement of their digital literacy. These factors may help explain why professors and associate professors exhibit higher levels of digital technology literacy.

When Table 9 is examined, it is observed that the differences in faculty members’ digital technology literacy levels and its sub-dimensions according to the variable of professional seniority are statistically significant (*p* < 0.01). *Post Hoc* Scheffe test results revealed that perceptions regarding “Digital Technology Literacy,” “Necessity of Digital Technology,” and “Self-Efficacy” were higher among those with 16–25 years and 26 years or more of seniority.

**Table 9:** ANOVA test results of faculty members’ digital technology literacy perception levels according to professional seniority variable.

Scale and sub-dimensions	Professional seniority	$\bar{X}$	*s*	*F*	*p*
Digital technology literacy	0–5 years	2.68	1.09	3.22	0.012*
	6–10 years	2.99	1.06		
	11–15 years	2.96	1.04		
	16–25 years	3.03	1.01		
	26 years and above	3.27	0.96		
Necessity of digital technology	0–5 years	2.61	1.1	4.303	0.002**
	6–10 years	3.05	1.16		
	11–15 years	3.17	1.14		
	16–25 years	3.22	1.15		
	26 years and above	3.2	1.07		
Self-efficacy	0–5 years	2.7	1.16	2.856	0.023*
	6–10 years	2.97	1.13		
	11–15 years	2.91	1.12		
	16–25 years	2.99	1.07		
	26 years and above	3.28	1.07		

*p < 0.05, *p < 0.01.

As faculty members gain more experience and seniority, the necessity to access diverse sources of information increases. In the digital age, utilizing digital technologies to access information has become an essential component of this need. The COVID-19 pandemic and the transition to remote education have compelled faculty members to interact intensively with technology (Keskin and Derya, 2020). This process has supported their ability to locate, utilize, and develop instructional materials, thereby enhancing the overall quality of education they deliver (Bakhov et al., 2021).

In the study by Baran et al. (2023), a significant difference was found between academic staff’s years of professional experience and their scores on the technology proficiency self-assessment scale. However, this difference indicated that technology proficiency scores tended to decrease as academic experience increased. Similar findings were reported in studies by Mücevher and Erdem (2018) and Fisher and Newton (2014). The literature suggests that as professional experience increases, faculty members encounter fewer difficulties in integrating digital technologies into educational processes, and that their digital skills significantly influence the effectiveness of technology use in education (Guillén-Gámez et al., 2022; Guoyuan et al., 2011; Maity et al., 2020; Orellana et al., 2021).

In the current research, it was determined that faculty members’ digital technology literacy levels and its sub-dimensions significantly varied based on professional seniority, with those having 16–25 years and 26 years or more of experience showing higher levels. Several reasons may account for this. Faculty members who have been in the profession for many years are likely to have had greater exposure to digital technologies and accumulated more practical experience in using them. Ongoing engagement with professional development and adaptation to technological change may have further strengthened their ability to use digital tools and resources effectively. Moreover, senior academics are often more involved in research and scholarly publishing, which requires intensive use of digital technologies and thus elevates their digital literacy. These factors help explain the higher levels of digital technology literacy and related perceptions among more experienced faculty members.

When Table 10 is examined, it is observed that the differences in faculty members’ digital technology literacy levels and its sub-dimensions are statistically significant according to their academic discipline (*p* < 0.01). The results of the Scheffe *post-hoc* tests conducted to determine the source of these differences reveal that faculty members working in the fields of basic and engineering sciences have higher levels of perceptions regarding “Digital Technology Literacy,” “Perceived Necessity of Digital Technology,” and “Self-Efficacy.”

**Table 10:** ANOVA test results of faculty members’ digital technology literacy perception levels according to the field of study variable.

Scale and sub-dimensions	Field of study	$\bar{X}$	*s*	*F*	*p*
Digital technology literacy	Health sciences	2.99	1.09	12.251	0.001*
	Social sciences	2.75	1.01		
	Basic and engineering sciences	3.36	0.92		
	Sports and fine arts	2.81	1.06		
Necessity of digital technology	Health sciences	2.99	1.14	11.357	0.001*
	Social sciences	2.83	1.17		
	Basic and engineering sciences	3.48	1.01		
	Sports and fine arts	2.94	1.17		
Self-efficacy	Health sciences	3.00	1.17	10.43	0.001*
	Social sciences	2.73	1.09		
	Basic and engineering sciences	3.33	1.01		
	Sports and fine arts	2.78	1.13		

*p < 0.01.

Üstündağ et al. (2017) reported that science teacher candidates had relatively high levels of digital literacy. In a study conducted by Koç and Albayrak (2020), it was found that academics working in science-related fields used technology more intensively. Based on these findings, it can be concluded that academics in fields such as engineering, architecture, and science make more frequent use of digital technologies.

The significant difference found in relation to the academic discipline variable in this study may be attributed to the nature of basic and engineering sciences, which inherently require greater integration with digital technologies. Faculty members in these fields are compelled to use digital tools, software, and technological solutions in their research and teaching activities, making them more familiar with digital technologies. Moreover, faculty in engineering and basic sciences are often encouraged to follow and implement technological innovations, which further strengthens their engagement with digital technologies.

### Findings and interpretation regarding the relationship between faculty members’ perceptions of digital epistemological beliefs and their perceptions of digital technology literacy

In this section of the study, a Pearson correlation analysis was conducted to examine the fifth sub-problem aligned with the purpose of the research: “Is there a relationship between faculty members’ perceptions of digital epistemological beliefs and their perceptions of digital technology literacy?” The results of the analysis are presented in Table 11.

**Table 11:** Examination of the relationship between faculty members’ perceptions of digital epistemological beliefs and digital technology literacy.

Variables		1	2	3	4	5	6	7	8	9
Digital epistemological belief (1)	*r*	1	0.801	0.600	0.582	0.869	0.646	0.614	0.597	0.564
	*p*		0.001**	0.001**	0.001**	0.001**	0.001**	0.001**	0.001**	0.001**
Source of digital epistemology (2)	*r*		1	0.504	0.497	0.531	0.482	0.509	0.508	0.463
	*p*			0.001**	0.001**	0.001**	0.001**	0.001**	0.001**	0.001**
Nature of digital epistemology (3)	*r*			1	0.322	0.335	0.323	0.404	0.375	0.375
	*p*				0.001**	0.001**	0.001**	0.001**	0.001**	0.001**
Scope of digital epistemology (4)	*r*				1	0.374	0.322	0.358	0.369	0.324
	*p*					0.001**	0.001**	0.001**	0.001**	0.001**
Accuracy of digital epistemology (5)	*r*					1	0.372	0.504	0.481	0.465
	*p*						0.001**	0.001**	0.001**	0.001**
Function of digital epistemology (6)	*r*						1	0.422	0.424	0.384
	*p*							0.001**	0.001**	0.001**
Digital technology literacy (7)	*r*							1	0.712	0.984
	*p*								0.001**	0.001**
Necessity of digital technology (8)	*r*								1	0.574
	*p*									0.001**
Self-efficacy (9)	*r*									1
	*p*									

**p < 0.01.

According to the results of the Pearson correlation analysis examining the relationship between faculty members’ digital epistemological belief perceptions and their digital technology literacy perceptions, a strong, positive, and statistically significant relationship was found between these two constructs (*r* = 0.614; *p* < 0.01). Among the subdimensions of digital epistemological beliefs, the strongest correlation with digital technology literacy was observed in the “source of digital epistemology” subdimension (*r* = 0.509; *p* < 0.01). Furthermore, the highest correlation between subdimensions was found between “source of digital epistemology” and “necessity of digital technology” (*r* = 0.508; *p* < 0.01).

This strong positive relationship can be explained by the fact that digital epistemological beliefs enable faculty members to benefit from digital technologies more effectively and consciously. Moreover, when supported by digital epistemological beliefs, digital technology literacy facilitates faculty members’ effective use of digital tools. The fact that the “source of digital epistemology” subdimension shows the strongest correlation with digital technology literacy indicates that faculty members have a high level of competence in evaluating the source of digital information, which forms the foundation for effectively utilizing digital technologies.

Additionally, the high correlation between “source of digital epistemology” and “necessity of digital technology” suggests that the faculty members’ skills in evaluating digital information sources strengthen their beliefs in the necessity and utility of digital technology in education.

These findings reveal that the relationship between digital epistemological beliefs and digital technology literacy is an interdependent process; as digital epistemological belief perceptions increase, digital technology literacy also tends to increase. This study makes a unique contribution by addressing a gap in the literature regarding this relationship.

### Findings and interpretation regarding the prediction of faculty members’ digital technology literacy perceptions by their digital epistemological belief perceptions

To examine the sixth sub-problem of the research “Do faculty members’ digital epistemological belief perceptions predict their digital technology literacy perceptions?” a regression analysis was conducted, and the results are presented in Table 12.

**Table 12:** Regression analysis on the relationship between faculty members’ digital epistemological belief perceptions and their digital technology literacy perceptions.

Variables	Β	s.h	*t*	*p*
Digital technology literacy	0.761	0.115	6.636	0.001**
Digital epistemological belief	0.715	0.035	20.172	0.001**

**p < 0.01; A linear regression analysis was conducted. F: 406.905; F(p): 0.001; Durbin-Watson: 1.983; R^2^: 0.377. Independent variable: Digital Epistemological Belief. Dependent variable: Digital Technology Literacy.

When the values in Table 12 are examined, it is determined that the perceived level of digital epistemological beliefs shows a strong and significant relationship with the perceived level of digital technology literacy (*β* = 0.715; *t* = 20.172; *p* < 0.01). This result indicates that a one-unit increase in digital epistemological beliefs would lead to a 0.715-unit increase in digital technology literacy. Furthermore, it was found that digital epistemological beliefs explain 37.7% of the variance in digital technology literacy (*R*^2^ = 0.377), and this level of explanatory power is considered adequate.

These findings suggest that digital epistemological beliefs may significantly influence digital technology literacy through factors such as the nature of knowledge, critical thinking, information management, learning and teaching approaches, and adaptability. These factors contribute to raising awareness about the source and accuracy of digital information, enhancing critical thinking and information management skills, increasing the integration of digital tools into educational processes, and facilitating adaptation to new technologies. Therefore, it can be stated that perceptions of digital epistemological beliefs significantly affect perceptions of digital technology literacy.

According to the data in Table 13, it was found that the subdimensions of digital epistemological beliefs have a significant effect on digital technology literacy levels. A one-unit improvement in the subdimensions of the source, nature, accuracy, and function of digital epistemology leads to increases of 0.173, 0.149, 0.275, and 0.167 units, respectively, in digital technology literacy. However, the effect of the scope dimension of digital epistemology was not found to be significant. These findings indicate that the subdimensions of digital epistemological beliefs explain 38.2% of digital technology literacy (*R*^2^ = 0.382). In particular, the impact of the “accuracy” subdimension highlights how the importance that faculty members place on the accuracy of information influences their digital literacy. While other subdimensions also significantly affect digital technology literacy, the “scope” of digital epistemology appears to have no effect. This suggests that digital technology literacy is more closely related to specific elements of digital epistemology, such as source, accuracy, and function.

**Table 13:** Regression analysis on the relationship between subdimensions of digital epistemological beliefs and digital technology literacy.

Variables	β	s.h	*t*	*p*	Tolerance	VIF
Digital technology literacy	0.710	0.117	6.049	0.001**		
Source of digital epistemology	0.173	0.042	4.019	0.001**	0.497	2.011
Nature of digital epistemology	0.149	0.029	4.181	0.001**	0.729	1.372
Scope of digital epistemology	0.068	0.028	1.891	0.059	0.726	1.377
Accuracy of digital epistemology	0.275	0.033	7.466	0.001**	0.684	1.461
Function of digital epistemology	0.167	0.031	4.700	0.001**	0.738	1.356

**p < 0.01; Multiple linear regression analysis was conducted. F: 82.331; F(p): 0.001; Durbin-Watson: 1.996; R^2^: 0.382. Independent variables: Source, Nature, Scope, Accuracy, Function. Dependent variable: Digital Technology Literacy.

The results demonstrate that digital epistemological beliefs have a strong impact on digital technology literacy and that this interaction should be taken into account to enhance faculty members’ digital literacy levels. Moreover, these findings increase the originality and significance of the study by addressing the gap in the literature regarding the effect of faculty members’ perceptions of digital epistemological beliefs on their perceptions of digital technology literacy.

## Conclusion

Findings related to the first sub-problem of the study show that faculty members’ digital epistemological beliefs are generally at a moderate level. The subdimensions—source, nature, scope, accuracy, and function of digital epistemology—were also evaluated at a moderate level. This result indicates a consistency and balance in faculty members’ digital epistemological beliefs, revealing that their perceptions of digital technologies and digital information are neither entirely positive nor negative. Furthermore, the moderate level of digital epistemological beliefs suggests that there is suitable potential for development in this area.

Results related to the second sub-problem indicate that faculty members’ perceptions of digital technology literacy are also at a moderate level. Their perceptions of the necessity of digital technology and their self-efficacy were likewise found to be moderate. This implies that faculty members acknowledge the importance of digital technology and feel confident in their digital skills, without showing excessive confidence or hesitation. Additionally, it demonstrates that faculty members possess the potential to use digital technologies effectively and that there is a solid foundation for further development. These findings emphasize the importance of training and support programs tailored to the needs of faculty members in order to enhance digital technology literacy.

The third sub-problem of the study examined how faculty members’ digital epistemological belief perceptions differ according to various variables. The results showed no significant differences in terms of gender. However, differences were observed based on academic title, professional seniority, and academic discipline. Faculty members with the titles of professor or associate professor, those with 16–25 years or more than 26 years of experience, and those working in the basic and engineering sciences fields have higher levels of digital epistemological belief perceptions. These groups tend to have a more positive attitude toward digital technology and information. These findings underscore the importance of customizing training programs to meet different needs in order to foster digital epistemological beliefs.

The fourth sub-problem explored how faculty members’ perceptions of digital technology literacy vary according to different variables. The results revealed no significant difference in terms of gender. However, differences were observed based on academic title, professional seniority, and academic discipline. Faculty members with the titles of professor or associate professor, those with 16–25 years or more than 26 years of experience, and those working in basic and engineering departments exhibited higher levels of digital technology literacy perceptions. These groups believe they are more competent in using and understanding digital technologies. These results highlight the necessity of customizing training and support programs based on title, seniority, and discipline, and stress that these factors should be considered when aiming to improve digital technology literacy.

According to the results of the Pearson correlation analysis, a strong and significant positive relationship was found between faculty members’ perceptions of digital epistemological beliefs and their perceptions of digital technology literacy. This indicates that as digital epistemological beliefs become stronger, levels of digital technology literacy also increase.

Based on the results of the regression analysis conducted to address the sixth sub-problem of the study, it was found that digital epistemological beliefs explain 37.7% of the variance in digital technology literacy. This suggests that digital epistemological beliefs significantly predict digital literacy. Furthermore, it was determined that the subdimensions of the source, nature, accuracy, and function of digital epistemology have significant effects on digital technology literacy, whereas the scope subdimension does not have a significant effect. These findings indicate that by strengthening their digital epistemological beliefs, faculty members can improve their digital literacy levels. Therefore, developing faculty members’ digital epistemological beliefs is essential for the effective use of digital technologies in education.

### Recommendations

This section presents recommendations developed for both practitioners and researchers based on the results of the study.

#### Recommendations for practitioners

- In order to support the digital technology competencies of faculty members—which is crucial for aligning higher education institutions with 21st-century digital pedagogy—awareness training can be provided to enhance faculty members’ digital epistemological beliefs.
- Recognizing the inevitability of digitalization, higher education institutions should offer internal training to enhance faculty digital competencies. The Council of Higher Education can support this by promoting practice-based programs focused on digital and information literacy. For example, the Presidential Digital Transformation Office provides free online courses on AI awareness and 21st-century skills via the “Uzaktan Eğitim Kapısı” (Remote Education Portal), platform. These existing models can inspire the development of new, targeted training initiatives.
- Courses or topics related to scientific epistemological beliefs can be integrated into faculty and college curricula.
- Courses or topics related to digital technology literacy can also be incorporated into higher education programs.
- It would be beneficial for universities to collaborate with institutions specialized in digital literacy and other relevant organizations.
- Individuals and institutions that produce knowledge and educate students must be able to access the correct sources of information. To achieve this, literacy skills related to knowledge must be developed, making the identification and enhancement of epistemological beliefs essential.
- Educational initiatives should be revised to meet the needs of the modern era and be more frequently integrated into teaching programs.
- In aligning higher education with the 21st century, international standards should be considered, and new policies and programs should be developed regarding digitalization.

#### Recommendations for researchers

- This study was conducted with faculty members from public universities. Future research could include both public and private university faculty to allow for comparative evaluations.
- Since this study was limited to domestic participants, similar research can be carried out internationally. Comparative analysis between domestic and international data could provide valuable insights.
- The independent variables in this study were gender, academic title, years of experience, and field of study. Future studies could explore additional variables for comparative analysis.
- A quantitative method was used in this research. Future research could adopt qualitative or mixed-method approaches to deepen understanding.
- Longitudinal research designs may be employed to examine the relationships between the variables more comprehensively.
- Future studies may benefit from incorporating multiple data sources in addition to self-report data collection instruments.
- Different methods and techniques may be utilized during the data collection process to reduce the possibility of common method bias.

## Data Availability

The original contributions presented in the study are included in the article/supplementary material, further inquiries can be directed to the corresponding author.

## Author’s note

This article is part of the first author's doctoral dissertation titled “Investigating The Relationship Between Academic Staff's Digital Epistemological Beliefs and Digital Technology Literacy” conducted under the supervision of the second author.
