# Supplementary material for: Digital epistemological beliefs as predictors of academics’ digital technology literacy
Source: Front Psychol. 2026 Jun 11;17:1848263. doi: 10.3389/fpsyg.2026.1848263 (PMC13293823; doi:10.3389/fpsyg.2026.1848263)
Supplement: Supplementary file 1 [file Supplementary_file_1.docx]

**APPENDICES**

**Appendix 1. Digital Epistemological Belief Scale**

(5 = Strongly Agree, 4 = Mostly Agree, 3 = Moderately Agree, 2 = Slightly Agree, and 1 = Strongly Disagree)

| **No** | **Digital Epistemological Belief Scale** | **5** | **4** | **3** | **2** | **1** |
| --- | --- | --- | --- | --- | --- | --- |
| 1 | The source of digital knowledge is reason. |  |  |  |  |  |
| 2 | Digital knowledge represents reality (truth). |  |  |  |  |  |
| 3 | The nature of digital knowledge is social. |  |  |  |  |  |
| 4 | Digital knowledge is the product of interaction between the human mind and machines. |  |  |  |  |  |
| 5 | The content of digital knowledge is scientific. |  |  |  |  |  |
| 6 | Digital knowledge is the sole form of knowledge in the Information Age. |  |  |  |  |  |
| 7 | The source of digital knowledge is experimentation and observation. |  |  |  |  |  |
| 8 | The source of digital knowledge is intuition. |  |  |  |  |  |
| 9 | The source of digital knowledge is intelligent systems such as artificial intelligence. |  |  |  |  |  |
| 10 | Digital knowledge is a new way of knowing. |  |  |  |  |  |
| 11 | The most valuable knowledge today is digital knowledge. |  |  |  |  |  |
| 12 | Digital knowledge is infinite. |  |  |  |  |  |
| 13 | Digital knowledge is the easiest source of information to access. |  |  |  |  |  |
| 14 | The correct answers to all scientific questions are found in digital knowledge. |  |  |  |  |  |
| 15 | Today, digital knowledge is the primary source in higher education. |  |  |  |  |  |
| 16 | Digital knowledge is one of the most frequently used sources of information among university students. |  |  |  |  |  |
| 17 | People are obliged to believe in digital knowledge. |  |  |  |  |  |
| 18 | Digital knowledge is always reliable. |  |  |  |  |  |
| 19 | Digital knowledge is universal. |  |  |  |  |  |
| 20 | All kinds of information can be found in digital information sources. |  |  |  |  |  |
| 21 | All forms of knowledge can be expressed through digital knowledge. |  |  |  |  |  |
| 22 | Digital knowledge is absolute. |  |  |  |  |  |
| 23 | Digital knowledge transcends time. |  |  |  |  |  |
| 24 | Digital knowledge is compatible with the epistemology of the Information Age. |  |  |  |  |  |

**Appendix 2. Digital Technology Literacy Scale**

(5 = Strongly Agree, 4 = Mostly Agree, 3 = Moderately Agree, 2 = Slightly Agree, and 1 = Strongly Disagree)

| **No** | **Digital Technology Literacy Scale** | **5** | **4** | **3** | **2** | **1** |
| --- | --- | --- | --- | --- | --- | --- |
| 1 | The use of digital technologies for educational purposes in higher education is necessary. |  |  |  |  |  |
| 2 | The use of digital technologies for learning purposes in higher education is necessary for students’ academic development. |  |  |  |  |  |
| 3 | Turkey/… is legally prepared for the use of digital technologies for learning purposes in higher education. |  |  |  |  |  |
| 4 | I have sufficient general knowledge about using digital technologies for learning purposes. |  |  |  |  |  |
| 5 | I have sufficient knowledge about the pedagogical theories and principles underlying the use of digital technologies for learning purposes. |  |  |  |  |  |
| 6 | I have sufficient knowledge and skills regarding communicating with students through different channels (chat, social media, e-mail, etc.) in online classroom management. |  |  |  |  |  |
| 7 | I have sufficient knowledge and skills regarding strategies for attracting students’ attention in online courses. |  |  |  |  |  |
| 8 | I have sufficient knowledge and skills regarding motivating students in online courses. |  |  |  |  |  |
| 9 | I have sufficient knowledge and skills regarding engaging students in online courses. |  |  |  |  |  |
| 10 | I have sufficient knowledge and skills regarding considering students’ individual differences in online courses. |  |  |  |  |  |
| 11 | I have sufficient knowledge and skills regarding selecting and using appropriate instructional strategies, methods, and techniques for online courses. |  |  |  |  |  |
| 12 | I have sufficient knowledge and skills regarding questioning strategies for students in online courses. |  |  |  |  |  |
| 13 | I have sufficient knowledge and skills regarding preparing and presenting digital instructional materials (presentations, digital stories, wikis, blogs, etc.) in online courses. |  |  |  |  |  |
| 14 | I have sufficient knowledge and skills regarding preparing lesson videos using multimedia elements (audio, visuals, text, etc.) in online courses. |  |  |  |  |  |
| 15 | I have sufficient knowledge and skills regarding using digital technologies to create educational and instructional materials in online courses. |  |  |  |  |  |
| 16 | I have sufficient knowledge and skills regarding downloading and installing software for hardware to be used in online courses via the internet or CD. |  |  |  |  |  |
| 17 | I have sufficient knowledge and skills regarding installing and using ready-made educational software (animations, simulations, tutorial packages, etc.) to prepare and implement digital lesson plans. |  |  |  |  |  |
| 18 | I have sufficient knowledge and skills regarding sharing my course materials as files through wireless networks (wireless, Bluetooth, infrared, etc.). |  |  |  |  |  |
| 19 | I have sufficient knowledge and skills regarding archiving all kinds of information and data in virtual environments (cloud, Google Drive, Dropbox, e-mail, etc.) over the internet in online courses. |  |  |  |  |  |
| 20 | I have sufficient knowledge and skills regarding formatting portable storage devices (disks) used in my online courses. |  |  |  |  |  |
| 21 | I have sufficient knowledge and skills regarding assigning homework to students in online courses. |  |  |  |  |  |
| 22 | I have sufficient knowledge and skills regarding measuring and evaluating students’ digital learning outcomes in online courses. |  |  |  |  |  |
| 23 | I have sufficient knowledge and skills regarding guiding students in online courses. |  |  |  |  |  |
| 24 | I believe that the online courses conducted during the COVID-19 period were effective and efficient. |  |  |  |  |  |
| 25 | I conducted my courses online during the COVID-19 period. |  |  |  |  |  |
